# Heterosexist Discrimination and Substance Use in Young Adult Sexual Minority Men: Examining the Moderating Role of Mindfulness

**DOI:** 10.1089/heq.2024.0015

**Published:** 2024-09-12

**Authors:** Dale Dagar Maglalang, Lance Keene, Fatima A. Mabrouk, Jasmine Agostino, Arryn A. Guy, Shufang Sun

**Affiliations:** ^1^ Silver School of Social Work, New York University, New York, New York, USA.; ^2^ Department of Health Services, Policy, and Practice, Brown University School of Public Health, Providence, Rhode Island, USA.; ^3^ Department of Behavioral and Social Sciences, Brown University School of Public Health, Providence, Rhode Island, USA.

**Keywords:** sexual minorities, cigarette use, e-cigarette use, alcohol, discrimination, mindfulness

## Abstract

**Purpose:**

Young sexual minority adults experience high rates of heterosexist discrimination. The use of substances has been documented as a form of coping with discrimination. While mindfulness has been used to address experiences of discrimination and the use of substances, respectively, few studies have explored whether mindfulness can lower the negative effects of discrimination on substance use. The purpose of this study is to examine the association between heterosexist discrimination and substance use in young adult sexual minority men (YASMM), and if dispositional mindfulness can moderate this relationship.

**Methods:**

Logistic regression analysis was used to examine the association of heterosexist discrimination and cigarette, e-cigarette, and hazardous drinking in a sample of YASMM (18–35 years old; *n* = 325) from a national survey. A two-way interaction analysis was also implemented to evaluate if dispositional mindfulness moderated this relationship.

**Results:**

Heterosexist discrimination was associated with increased odds of cigarette use (adjusted odds ratio [aOR] = 1.06; 95% confidence interval [CI]: 1.03, 1.08) and e-cigarette use (aOR = 1.03; 95% CI: 1.01, 1.05). Higher scores of dispositional mindfulness moderated the relationship between heterosexist discrimination and hazardous drinking, indicating a weakening effect with higher scores of dispositional mindfulness.

**Conclusion:**

Mindfulness may decrease the negative effects of heterosexist discrimination on hazardous drinking among YASMM.

Health Equity Implications: Researchers and practitioners should consider incorporating mindfulness as a component to their intervention to help YASMM deal with stressors engendered by discrimination, which may prevent hazardous drinking as a coping mechanism at a younger age.

## Introduction

Sexual minority (SM) young adults report experiencing high rates of heterosexism (e.g., sexual orientation-based rejection, harassment, and discrimination),^1^ which refers to a system that provides a range of systematic advantages to heterosexual individuals that are reinforced through interpersonal, institutional, and systematic discriminatory experiences for sexually minoritized individuals (i.e., SM young men).^2–5^ SM young adults are particularly vulnerable to discriminatory experiences given that young adulthood is a critical developmental period comprising important milestones and life events (e.g., cognitive, emotional, and behavioral development).^6^

Growing evidence has documented that experiences of heterosexist discrimination are linked to internalizing and externalizing behaviors that may contribute to a range of psychosocial problems,^7^ including the misuse of substances such as tobacco, nicotine, and alcohol as coping mechanisms in response to instances of discrimination related to their minoritized identities.^8,9^ Findings from national data on tobacco, nicotine, and alcohol use consistently show higher prevalence among SM than their heterosexual peers. Results from a 2021 national survey reveal that SM adults age 18 and older had higher prevalence rates of cigarette use (15.3% vs. 11.4%) and e-cigarette use (13.2% vs. 4.1%) compared with heterosexual adults.^10^ Similarly, binge alcohol use in 2021–2022 was also higher in gay men (31.1%) than heterosexual men (27.2%).^11^

The Minority Stress Theory (MST) provides a framework for understanding the process of how discrimination influences the use of substances. Virginia Brooks first introduced the concept of “minority stress” in her pioneering research on lesbian women. Brooks described it as a state resulting from the cumulative impact of culturally imposed inferior status, prejudice, and discrimination, which affect an individual’s cognitive framework that can lead to difficulties in adaptation.^12^ Brooks outlined four interrelated dimensions of minority stress: (1) cultural—feelings of inferiority associated with one’s identity, (2) social and economic—experiences of stigma, discrimination, and limited access to resources, (3) psychological—impacts on self-esteem and a sense of security, and (4) biophysical—effects of prolonged stress.^12^ According to the MST, individuals of minoritized community or social status (e.g., sexual orientation, race, gender) encounter identity-based discrimination and prejudice, leading to the internalization of stressors (e.g., internalized homophobia) and thereby contributing to negative mental health outcomes.^4,5,13^ While the primary focus of MST centers on mental health, empirical studies have extended its applicability to encompass substance-use outcomes.^14–16^

These studies have found that SMs experience sexual orientation- and gender identity-based stressors linked to victimization, lack of supportive environments, and psychological stress that predict the use of substances.^14,15^ Moreover, a range of studies demonstrate that SM individuals indicate greater rates of substance use than their heterosexual counterparts due to internalized heterosexism (IH).^17^ For example, in a multilevel meta-analysis designed to elucidate the relationship between IH and substance-use outcomes among diverse SM individuals, greater levels of IH were linked to increased use of substances, including alcohol, tobacco, heroin, and cocaine.^17^ Furthermore, while limited studies address the role of IH and smoking among SM, Kraus and colleagues^18^ found that the use of cigarettes and e-cigarettes was greater among bisexual, pansexual, and queer-identified individuals who were assigned male sex at birth, consistent with the MST.

Mindfulness as a behavioral health intervention has been emerging as a potential therapeutic approach to address poor mental health and substance misuse.^19,20^ The practice of mindfulness is derived from Buddhism and is defined as, “a particular way of paying attention, a process of non-judgmental awareness and an attitude of openness and acceptance.”^21^ Mindfulness may offer the benefits of alleviating the harmful effects of discrimination by increasing emotional awareness. For instance, individuals’ practice with present awareness may increase their ability to confront and process difficult emotions related to instances of discrimination by reducing reactivity and internalization of such experiences.^22^

Specific to SM populations, mindfulness may also help affirm sexual orientation, gender, and other intersecting identities, engender self-empowerment, and develop skills to enhance resilience and self-regulation.^23^ Moreover, studies demonstrate that individuals who report greater levels of mindfulness experience less mental distress when they encounter discrimination (e.g., homophobia and racism).^22,24^ While mindfulness has been used as an intervention tool to address substance misuse,^25^ there may be potential benefits of mindfulness for young adult sexual minority men (YASMM) since no research has examined the potential moderating role of mindfulness in mitigating the adverse effects of discrimination on substance misuse with this population.

Therefore, we aim to examine the association between heterosexist discrimination and use of cigarettes, e-cigarettes, and hazardous drinking among YASMM. In addition, we plan to assess if dispositional mindfulness, defined as the inherent ability to maintain attention on present-moment experiences with an open and nonjudgmental attitude, moderates this relationship.^26^ Our first hypothesis (H1) was higher scores of heterosexist discrimination would be associated with higher odds of use of the aforementioned substances. Our second hypothesis (H2) was dispositional mindfulness would moderate the relationship between heterosexist discrimination and substance use, such that this relationship weakens with higher scores of mindfulness. The findings from this study will provide evidence for leveraging mindfulness as an intervention with potential utility for YASMM populations who experience heterosexist discrimination and use substances.

## Methods

### Setting and Population

We conducted an internet-based survey between June 2021 and May 2022. Recruitment was made through paid Facebook and Instagram advertisements and in partnership with Lesbian, Gay, Bisexual, Transgender, Queer Plus (LGBTQ+) organizations in the United States. An example advertisement included a pictured hand with a rainbow behind the text, “There is HOPE, and You can Help! Participate in a Research Survey on Gay, Bisexual, and Queer Men’s Mental Health. Participate in online survey and earn $15.” We targeted our paid Facebook and Instagram advertisements to recruit participants from large urban centers, small urban areas, and rural counties. Using the Movement Advancement Project Equality Map, we organized states into “high,” “medium,” “fair,” “low,” and “negative” overall policy tally groups. From states listed in each of the policy tally levels, we then randomly selected using excel “RANDBETWEEN” function the following: three most populous cities for a total of 15 unique cities from differing states; three small urban areas (i.e., cities with a population of 100,000 or more, excluding the 10 most populous cities) for a total of 15 unique small urban areas from differing states; and three rural counties (i.e., counties with a population of 250,000 or fewer) for a total of 15 unique counties. We targeted our social media advertisements in these 45 areas. In terms of LGBTQ+ organizations, we contacted more than 70 community-based organizations with at least one organization per state in the United States. We also contacted more than 600 university-based LGBTQ+ organizations with representation from every state to disseminate recruitment efforts.

As the study had an aim of understanding and addressing HIV risk, the inclusion criteria included 18–35-year olds, able to read and write in English, live in the United States, assigned male at birth, HIV negative or unknown status, and ever had sex with a man. The total number of participants who completed the screener was 6,169; out of this sample, 2,925 completed the survey, 1,081 completed the postscreener, and 325 passed the manual review for data integrity. The final analytic sample was *n* = 325 YASMM. Of the final analytic sample, 67% (*n* = 219) came from community organization referrals, 20% (*n* = 64) from Facebook/Instagram paid advertisements, 8% (*n* = 26) from university organizations, and 5% (*n* = 16) from private LGBTQ+ Facebook groups. The screener and postsurvey screener were used for purposes of verifying data integrity. Data for this study are drawn from the main survey. A detailed description of our bot and data integrity review procedures is published here (article under review-masked peer review link of OSF page: https://osf.io/xydjk/?view_only=5694012cd33a48c3b3e8a5a625518593).

Eligible participants provided consent and completed the survey using Qualtrics XM platform. Survey items included questions on demographics, health behaviors, and mental health measures. Participants who completed the survey received a $15 Amazon gift card. The Institutional Review Board of Brown University reviewed and approved the study (protocol #2698).

### Measures

#### Dependent variables

##### Past 30-day cigarette and e-cigarette use

Two different questions were used to measure past 30-day cigarette and e-cigarette use from the Monitoring the Future Study.^27^ Participants were asked, “How often have you smoked cigarettes in the past 30 days” and “how often have you smoked e-cigarettes in the past 30 days?” respectively. Response categories were daily, less than daily but at least 2–3 times weekly, occasionally (less than 2–3 times weekly), and never in the past 30 days. We created a binary variable for using cigarettes/e-cigarettes in the past 30 days by collapsing the categories: “daily,” “less than daily,” and “occasionally” into 1 = any use in the past 30 days, and “never” into 0 = no use in the past 30 days. Previous studies have used binary versions of the variable to determine past 30-day cigarette^28^ and e-cigarette use.^29^ Past 30-day cigarette and e-cigarette use has been found to be a useful measure in predicting nicotine dependence and long-term use.^30,31^

##### Hazardous drinking

Hazardous drinking was measured using the Alcohol Use Disorders Identification Test-Concise (AUDIT-C), which is a screening measure to indicate risk for hazardous drinking or potential alcohol-use disorder (AUD).^32,33^ Hazardous drinking is conceptualized as the amount of and pattern of consuming alcohol that impose a health risk to individuals.^34^ The three-item measure asks participants, “How often do you have a drink containing alcohol” (never—4 or more times a week); “how many standard drinks containing alcohol do you have on a typical day” (1 or 2–10 or more); and “how often do you have six or more drinks on one occasion?” (daily or almost daily—never). Responses are scored using the total score ranging from 0 to 2. A cutoff score of 4 or more for men and 3 or more for women is considered engaging in hazardous drinking.^35^

#### Independent variable

Heterosexist discrimination was measured using the Heterosexist, Harassment, Rejection and Discrimination Scale,^36^ which is a 14-item scale that evaluates discrimination related to sexual orientation. Questions in the measure include, “How many times have you been rejected by friends because you are an LGBTQ person” and “how many times have you been verbally insulted because you are an LGBTQ person?” Response categories were rated using a 6-point Likert scale ranging from 1 = If the event has NEVER happened to you to 6 = If the event happened ALMOST ALL OF THE TIME (more than 70% of the time). Higher scores indicate more experiences of heterosexist discrimination. The internal consistency of the scale in the current sample was α = 0.94.

#### Moderator variable

Dispositional mindfulness was measured using the Mindful Attention Awareness Scale, which is a 15-item scale that evaluates the frequency of mindfulness in everyday experiences.^26^ Statements in the measure include, “I find it difficult to stay focused on what’s happening in the present” and “I rush through activities without being really attentive to them.” Response categories were rated using a 6-point Likert scale ranging from 1 = almost always to 6 = almost never. Higher scores indicate more frequent experiences of receptive awareness and perception in the present. An average score of the responses is used to determine dispositional mindfulness. The internal consistency of the scale was α = 0.93.

### Covariates

Covariates included in the model were age, race/ethnicity (non-Hispanic [NH] White, Hispanic, NH Black, Indigenous, and People of Color [BIPOC]), education (some high school [HS], general education degree [GED], some college), religiously affiliated, and mental health. Mental health was measured using the mental health subscale (4 items) of the Patient-Reported Outcomes Measurement Information System.^37^ Participants reported their general quality of life, mental health, social satisfaction, and their past-week emotional difficulties using a 5-point Likert scale ranging from 1 = always to 5 = never. A sum of the scores was calculated. The internal consistency of the scale was α = 0.66, which is deemed to be in the acceptable range.^38^

We adjusted for the covariates because these variables are known confounders of the independent and dependent variables. For instance, poor mental health has been associated with experiences of discrimination^39^ and use of substances such as tobacco, nicotine, and alcohol,^40^ respectively. Similarly, religious affiliation has also been found to be positively or negatively associated with discrimination,^41,42^ substance use,^43^ and mindfulness,^44^ respectively, depending on the population and context. Previous literature has stated that adjustment for confounders should also include the moderator because it can lead to a misspecification of the model.^45^ Religious affiliation was measured with the question, “How do you describe your religion, spiritual practice, or existential worldview?” Response categories were extensive and included options such as Buddhist, Christian, Hindu, Jewish, and Muslim. Participants who identified with a religion or spiritual practice were categorized as 1 = Religiously Affiliated.

### Statistical Analysis

Univariate analysis was used to evaluate the distribution of the variables descriptively. Subsequently, we used logistic regression to examine associations between heterosexist discrimination and cigarette use, e-cigarette use, and hazardous drinking, respectively, adjusting for the moderator variable and covariates. We then implemented a two-way interaction to examine the moderating role of dispositional mindfulness between heterosexist discrimination and the use of substances.

Heterosexist discrimination and dispositional mindfulness were mean-centered for the moderation analysis for ease of interpretation and to reduce multicollinearity.^46^ We used dispositional mindfulness as a moderator because it is not in the causal pathway of the independent and dependent variables.^47^ Furthermore, the purpose of the study is to examine whether levels of dispositional mindfulness (low vs. high scores) change the relationship between heterosexist discrimination and the use of substances, in which moderation analysis is the appropriate data analysis approach.^47^ Other studies have used dispositional mindfulness as a moderator.^48,49^ Finally, an omnibus Wald test was implemented to understand if the overall interaction was statistically significant. Stata 17.0 was used to analyze the data.

### Sensitivity Analysis

Since past 30-day cigarette and e-cigarette use were measured using ordinal categories and acknowledging that there are clinically meaningful differences between participants who use cigarettes and e-cigarettes never, occasionally, less than daily, and daily in the past 30 days, we implemented a sensitivity analysis. The categories of occasionally and less than daily were combined because of the low number of observations in each cell. First, to determine whether to analyze the data using ordinal logistic regression or multinomial logistic regression, we used the O model package in Stata.^50^ The analysis showed that the proportional odds assumptions were violated for both models of cigarette and e-cigarette use (Prob>chi2 = <0.0001). As a result, the data were analyzed using multinomial logistic regression. We analyzed the AUDIT-C measure as a continuous variable using linear regression. Sensitivity analysis results are reported in Supplementary Tables S1, S2, S3, and S4.

## Results

Table 1 displays the demographic characteristics of the participants. Of the *n* = 325 YASMM, 51% used cigarettes in the past 30 days, 36% used e-cigarettes in the past 30 days, and 38% engaged in potentially hazardous drinking. The mean reported experience of heterosexist discrimination was mean *[M]* = 37.90 (standard deviation [*SD*] = 12.05; range: 14–73), and dispositional mindfulness was *M* = 3.66 (*SD* = 0.89; range: 1.8–6). The mean age of the participants was 27 years (*SD* = 3.91). The sample was predominantly NH White (51%), had a college degree or higher (59%), religiously affiliated (62%), and the mean reported overall mental health was *M* = 13.09 (*SD* = 2.28; range: 6–20).

**Table 1.** Demographic Characteristics of Young Adult Sexual Minority Men (*n* = 325)

	Obs. % or *M*(*SD*)
Heterosexist discrimination (14–73)^a^; mean centered	37.90 (12.05); −1.58e-07 (12.05)
Dispositional mindfulness (1.8–6)^a^; mean centered	3.66 (0.89); 1.12e-09 (0.89)
Past 30-day cigarette use	166 (51.08%)
Past 30-day e-cigarette use	117 (36%)
Hazardous drinking^b^	122 (37.54%)
Age (18–35)	26.68 (3.91 years)
Race/ethnicity	
Non-Hispanic White	165 (50.77%)
Hispanic and Non-Hispanic BIPOC	160 (49.23%)
Education	
Some high school/GED/some college	132 (40.62%)
College/graduate degree	193 (59.38%)
Religiously affiliated	201 (61.85%)
Mental health (6–20)^a^	13.09 (2.28)

^a^ Range of reported values of the sample.

^b^ Hazardous drinking is defined as a score of 4 or more on the Alcohol Use Disorders Identification Test-Concise (AUDIT-C).

Obs., observation; M, mean; SD, standard deviation; BIPOC, Black, Indigenous, and People of Color; GED, general education development.

### Main effects of heterosexist discrimination on substance use and hazardous drinking

Table 2 displays the logistic regression analysis of heterosexist discrimination and the use of substances. Experiences of heterosexist discrimination were associated with increased odds of cigarette use (adjusted odds ratio [aOR] = 1.06; 95% confidence interval [CI]: 1.03, 1.08) and e-cigarette use (aOR = 1.03; 95% CI: 1.01, 1.05). The association between heterosexist discrimination and hazardous drinking was not significant (aOR = 1.01; 95% CI: 0.99, 1.03).

**Table 2.** Logistic Regression Analysis of Heterosexist Discrimination and Use of Substances (*n* = 325)

	Past 30-day cigarette use aOR (95% CI)	Past 30-day e-cigarette use aOR (95% CI)	Hazardous drinking aOR (95% CI)
Heterosexist discrimination	1.06 (1.03, 1.08)^***^	1.03 (1.01, 1.05)^**^	1.01 (0.99, 1.03)
Dispositional mindfulness	1.46 (1.08, 1.96)^*^	1.32 (0.99, 1.77)	0.74 (0.55, 1.00)
Age	1.01 (0.94, 1.09)	1.04 (0.96, 1.11)	0.91 (0.85, 0.98)^*^
Hispanic and Non-Hispanic BIPOC (reference Non-Hispanic White)	3.58 (2.09, 6.12)^***^	2.19 (1.27, 3.76)^**^	2.52 (1.50, 4.26)^**^
College/Graduate degree (reference some HS/GED/some college)	0.90 (0.51, 1.61)	1.76 (0.99, 3.14)	1.36 (0.77, 2.41)
Religiously affiliated (reference no religious affiliation)	2.45 (1.45, 4.12)^**^	0.44 (0.26, 0.74)^**^	1.69 (1.00, 2.86)
Mental health	1.10 (0.98, 1.23)	0.90 (0.80, 1.00)	0.96 (0.85, 1.07)

^*^ *p* < 0.05.

^**^ *p* < 0.01.

^***^ *p* < 0.001.

aOR, adjusted odds ratio; CI, confidence interval.

### The Interaction Effects of Mindfulness × Heterosexist Discrimination

**FIG. 1.**
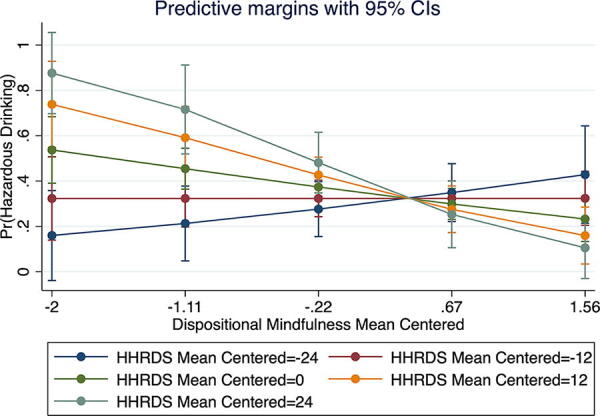
Associations between heterosexist discrimination and hazardous drinking by dispositional mindfulness. HHRDS, Heterosexist, Harassment, Rejection, and Discrimination Scale; Pr, probability.

Dispositional mindfulness did not have a moderating effect on the relationship between heterosexist discrimination and cigarette use (aOR = 1.00; 95% CI: 0.97, 1.03) and e-cigarette use (aOR = 0.98; 95% CI: 0.96, 1.01). However, dispositional mindfulness moderated the relationship between heterosexist discrimination and hazardous drinking (aOR = 0.97; 95% 0.94, 0.99; Table 3), indicating a weakening effect with higher scores of dispositional mindfulness (Fig. 1). The interaction term was statistically significant [χ^2^(3) = 10.49, *p* < 0.01]. The main effect of mindfulness was also significant, such that those with higher levels of dispositional mindfulness had lower odds of engaging in hazardous drinking (aOR = 0.65; 95% CI: 0.47, 0.91).

**Table 3.** Interaction Term of the Moderating Role of Dispositional Mindfulness on Heterosexist Discrimination and Hazardous Drinking (*n* = 325)

	Past 30-day cigarette use aOR (95% CI)^a^	Past 30-day e-cigarette use aOR (95% CI)^a^	Hazardous drinking aOR (95% CI)^a^
Heterosexist discrimination	1.06 (1.03, 1.08)^***^	1.03 (1.01, 1.06)^**^	1.01 (0.99, 1.04)
Dispositional mindfulness	1.46 (1.07, 1.99)^*^	1.28 (0.95, 1.72)	0.65 (0.47, 0.91)^*^
Heterosexist discrimination × Dispositional mindfulness	1.00 (0.97, 1.03)	0.98 (0.96, 1.01)	0.97 (0.94, 0.99)^*^

^a^ Models were adjusted with the covariates (age, race/ethnicity, education, religious affiliation, and mental health).

^*^ *p* < 0.05.

^**^ *p* < 0.01.

^***^ *p* < 0.001.

### Sensitivity Analysis Results

The findings from the sensitivity analysis are consistent with the primary analysis that appraised the data with dichotomous outcomes. Experiences of heterosexist discrimination were associated with occasionally/less than daily (relative risk ratio [RRR] = 1.10; 95% CI: 1.06, 1.14) and daily using cigarettes (RRR = 1.04; 95% CI: 1.01, 1.07) and occasionally/less than daily (RRR = 1.03; 95% CI: 1.00, 1.05) and daily using e-cigarettes (RRR = 1.07 95% CI: 1.01, 1.14) relative to never using cigarettes and e-cigarettes in the past 30 days, respectively (Supplementary Table S2). Similarly, higher scores of dispositional mindfulness moderated the relationship between experiences of heterosexist discrimination and hazardous drinking (b = −0.03, 95% CI: −0.05, −0.002; Supplementary Table S4) but not for past 30-day cigarettes and e-cigarette use. Thus, these findings may suggest that for YASMM participants in this study, experiencing heterosexist discrimination may not necessarily influence the more expansive categorical and continuous variability of using cigarettes and e-cigarettes and engaging in hazardous drinking in the past 30 days.

## Discussion

This study sought to examine the association of heterosexist discrimination and the use of substances among YASMM and assess if dispositional mindfulness moderated the aforementioned relationship. Experiences of heterosexist discrimination were associated with increased odds of cigarette and e-cigarette use but not hazardous drinking. Dispositional mindfulness moderated the association between heterosexist discrimination and hazardous drinking, demonstrating reduced odds of engaging in hazardous drinking relevant to its associations with experiences of heterosexist discrimination for those with higher scores of dispositional mindfulness.

The findings are concordant with previous studies on heterosexist discrimination and the use of substances in SMs.^51,52^ As proposed by the MST,^4,13^ experiences of stressors such as discrimination related to one’s sexual orientation and gender identity can contribute to substance use in SMs.^15^ YASMM are particularly vulnerable to experiences of heterosexist discrimination as young adulthood is an important phase in their lives where critical life stages and developments occur.^6^ Experiencing heterosexist discrimination can directly lead to substance use as a form of stress-related coping, or as shown in previous research, through other mechanisms such as internalized homophobia.^15^

Studies also show that early onset of cigarettes and alcohol at a younger age has been associated with long-term use into adulthood and concomitant use of other substances,^30,53,54^ making this life stage critical for substance-use prevention and intervention that may provide lifelong benefits. Notably, Latine/x and NH BIPOC YASMM navigate a range of additional intersectional stressors (e.g., racism, heterosexism, sexism) that may influence substance use (e.g., cigarette, e-cigarette, alcohol use) and mental health outcomes.^55,56^ This can have critical implications since SMs have less access to substance-use treatment compared with their heterosexual counterparts.^57^

Higher scores of dispositional mindfulness reported by YASMM moderated the relationship between heterosexist discrimination and hazardous drinking but not cigarette and e-cigarette use. Participants may have different preferences in their choice of substances as a coping mechanism when experiencing stressors related to heterosexist discrimination. While the use of substances such as cigarette and alcohol can be co-occurring, perhaps some participants in the study preferred to use one substance, specifically, alcohol, rather than cigarettes or e-cigarettes, and that participants who use alcohol may report higher scores of dispositional mindfulness than those who use cigarettes and e-cigarettes as a form of coping mechanism. Furthermore, it may also be a result of differences in the intensity of the use of these substances to the extent that participants who reported higher scores of dispositional mindfulness who engage in hazardous drinking are drinking less frequently within the span of the past 30 days than someone who may use cigarettes and e-cigarettes more frequently in the same time frame because of reasons such as convenience and affordability. Thus, dispositional mindfulness may have more of an effect for those who still use substances but are engaging in it less intensely or frequently and are still categorized in engaging in hazardous drinking when they do drink alcohol. The use of mindfulness as an intervention in reducing AUD is still emerging and has shown promising results that it is on par with existing AUD psychosocial treatments.^58^ Mindfulness may be effective in addressing stress associated with heterosexist discrimination that influences hazardous drinking because it helps individuals accept stressful experiences and develop healthy coping mechanisms rather than using alcohol to numb their emotions.^58,59^

The main effects showed that dispositional mindfulness was associated with increased odds of past 30-day cigarette use. The findings are conflicting with other studies that found that mindfulness reduces smoking overtime.^60^ A potential explanation for this is that while mindfulness can help affirm people’s identities such as their sexual orientation,^23^ the concept of identity-relevant stressor^61^ suggests that having strong associations with one’s identity can also lead some individuals to experience greater stress when the identity they strongly identify with is being discriminated. Consequently, participants who reported higher scores of dispositional mindfulness may also make them feel more stressed if they are experiencing discrimination and their choice of coping from these stressful events may be smoking cigarettes rather than e-cigarettes and alcohol.

### Limitations

This study is not without its limitations. While the study disseminated the survey nationally, it used a convenience sampling approach, which does not make the YASMM sample representative of YASMM in the United States. Furthermore, this study is only limited to YASMM. Given the diversity of SMs, studies might benefit from examining how mindfulness may play a role in helping other SMs cope with heterosexist discrimination. The measurement of the outcome variables was measured dichotomously, although validated from previous studies, which has its limitations in demonstrating the variability and nuance of substance use. However, we conducted a sensitivity analysis that was consistent with the binary outcomes. Finally, the study was a cross-sectional survey, and causality cannot be concluded. Given the emerging studies on the use of mindfulness as a tool in reducing the use of substances, future studies should consider implementing a longitudinal intervention design to develop and evaluate mindfulness practices in YASMM who use substances to reduce their use.

## Conclusion

Heterosexist discrimination was associated with cigarette and e-cigarette use in a sample of YASMM. Dispositional mindfulness moderated the relationship between experiences of heterosexist discrimination and hazardous drinking but not for cigarette and e-cigarette use. Implications for researchers and practitioners need to consider how to help SMs, especially YASMM, develop tailored mindfulness practices in relation to experiences of heterosexist discrimination and their use and preference for different types of substances as a coping mechanism.

## Abbreviations Used

AUD: alcohol-use disorder
AUDIT-C: Alcohol Use Disorders Identification Test-Concise
BIPOC: Black, Indigenous, and People of Color
IH: internalized heterosexism
LGBTQ+: Lesbian, Gay, Bisexual, Transgender, Queer Plus
MST: Minority Stress Theory
NH: non-Hispanic
SM: sexual minority
YASMM: young adult sexual minority men
