# Supplementary material for: Heterosexist Discrimination and Substance Use in Young Adult Sexual Minority Men: Examining the Moderating Role of Mindfulness
Source: Health Equity. 2024 Sep 12;8(1):578–87. doi: 10.1089/heq.2024.0015 (PMC11464818; doi:10.1089/heq.2024.0015)
Supplement: Supplementary Table S1 [file heq.2024.0015_supplement.pdf]

Supplementary Table 1. Distribution of past 30 day cigarette and e-cigarette use as ordinal variables and hazardous drinking as a continuous variable (*N*=325).

	Obs. % or *M*(*SD*)
Past 30 day cigarette use	
Never in the past 30 days	159 (48.92%)
Occasionally/Less than daily	53 (16.31%)
Daily	113 (34.77%)
Past 30 day e-cigarette use	117 (36%)
Never in the past 30 days	208 (64%)
Occasionally/Less than daily	90 (27.69%)
Daily	27 (8.31%)
Hazardous drinking (0-9)[a]	2.91 (2.18)

Note. a. Range of reported values of the sample.

Supplementary Table 2. Multinomial logistic regression analysis of heterosexist discrimination and past 30 day use of cigarettes and e-cigarettes (*N*=325).

	Past 30 day cigarette use (ref Never in the past 30 days)		Past 30 day e-cigarette use (ref Never in the past 30 days)	
	Occasionally/Less than Daily RRR (95% CI)	Daily RRR (95% CI)	Occasionally/Less than Daily RRR (95% CI)	Daily RRR (95% CI)
Heterosexist discrimination	1.10 (1.06, 1.14)***	1.04 (1.01, 1.07)**	1.03 (1.00, 1.05)*	1.07 (1.01, 1.14)*
Dispositional mindfulness	1.19 (0.75, 1.88)	1.53 (1.11, 2.11)**	0.74 (0.51, 1.09)	7.48 (3.19, 17.57)***
Age	1.02 (0.92, 1.13)	1.00 (0.93, 1.09)	1.05 (0.98, 1.14)	0.99 (0.80, 1.22)
Hispanic and Non-Hispanic BIPOC (reference Non-Hispanic white)	3.42 (1.58, 7.39)**	3.71 (2.08, 6.62)***	1.84 (1.02, 3.33)*	1.63 (0.39, 6.74)
College/Graduate degree (reference Some HS/GED/Some college)	0.64 (0.30, 1.38)	1.04 (0.55, 1.95)	1.32 (0.73, 2.40)	8.56 (0.86, 85.36)
Religiously affiliated (reference no religious affiliation)	2.14 (1.01, 4.55)*	2.60 (1.46, 4.62)**	0.74 (0.42, 1.33)	0.08 (0.02, 0.37)**
Mental health	1.05 (0.88, 1.25)	1.12 (0.98, 1.27)	0.92 (0.81, 1.05)	0.76 (0.56, 1.04)

Note. *p < .05 **p < .01 ***p < .001. Abbreviations: Relative Risk Ratio (RRR); Confidence Interval (CI).

Supplementary Table 3. Linear regression analysis of heterosexist discrimination and hazardous drinking (*N*=325).

	b[a]	95% CI	β[b]
Heterosexist discrimination	0.0004	-0.02, 0.02	0.002
Dispositional mindfulness	-0.05	-0.32, 0.22	-0.02
Age	-0.04	-0.11, 0.03	-0.08
Hispanic and Non-Hispanic BIPOC (reference Non-Hispanic white)	1.21***	0.71, 1.72	0.28
College/Graduate degree (reference Some HS/GED/Some college)	0.13	-0.41, 0.66	0.03
Religiously affiliated (reference no religious affiliation)	0.24	-0.25, 0.73	0.05
Mental health	-0.03	-0.14, 0.07	-0.03

Note. *p < .05 **p < .01 ***p < .001. a. Unstandardized coefficient. b. Standardized coefficient. Abbreviations: Confidence Interval (CI).

Supplementary Table 4. Interaction term of the moderating role of dispositional mindfulness on heterosexist discrimination and hazardous drinking (*N*=325).

	$b^{a,b}$	95% CI	β^{c}
Heterosexist discrimination	0.003	-0.02, 0.02	0.02
Dispositional mindfulness	-0.13	-0.42, 0.15	-0.06
Heterosexist discrimination x Dispositional mindfulness	-0.03*	-0.05, -0.002	-0.12

Note. *p < .05 **p < .01 ***p < .001. a. Unstandardized coefficient. b. Model was adjusted with the covariates (age, race/ethnicity, education, religious affiliation, and mental health). c. Standardized coefficient. Abbreviation: Confidence Interval (CI).
